# Factors associated with treatment type of non-malarial febrile illnesses in under-fives at Kenyatta National Hospital in Nairobi, Kenya

**DOI:** 10.1371/journal.pone.0217980

**Published:** 2019-06-13

**Authors:** Borna A. Nyaoke, Marianne W. Mureithi, Caryl Beynon

**Affiliations:** 1 The University of Liverpool, Liverpool, United Kingdom; 2 The University of Nairobi, Nairobi, Kenya; Northwestern University Feinberg School of Medicine, UNITED STATES

## Abstract

**Background:**

Non-malarial febrile illnesses comprise of almost half of all fever presenting morbidities, among under-five children in sub-Saharan Africa. Studies have reported cases of prescription of antimalarial medications to these febrile under-fives who were negative for malaria. The treatment of these children with antimalarial medications increases incidences of antimalarial drug resistance as well as further morbidities and mortalities, due to failure to treat the actual underlying causes of fever.

**Aim:**

To identify clinical and demographic factors associated with treatment type (malarial/non-malarial) of non-malarial febrile illnesses (NMFI) in children aged ≤5 at the Kenyatta National Hospital in Nairobi, Kenya.

**Methods:**

A positivist epistemological approach, cross sectional descriptive study design was used. A structured questionnaire was used on a sample of 341 medical records of children aged ≤5 years to extract data on clinical examinations (recorded as yes or no), diagnostic test results, and demographic data on the child’s sex and age. Descriptive and inferential analysis was applied to the data.

**Results:**

Prescription of antimalarial drugs despite negative microscopy results was found in 44 (12.9%) of the children, with mortality reported in 48 (14.1%). Assessment of respiratory distress was 0.13 (0.03,0.58) times associated with less likelihood of prescribing an antimalarial in those with a negative microscopy. A male patient was 0.21 (0.05,0.89) times less likely to receive an intravenous antimalarial after a negative microscopy. Patients aged ˂1 with a negative microscopy result were more likely to receive an antimalarial than older children.

**Conclusion:**

There is a need to eliminate incorrect treatment of NMFI with antimalarial medication, while ensuring correct diagnosis and treatment of the specific illness occurs. This requires strengthening and adherence to diagnostic and treatment guidelines of febrile illnesses in under-fives, consequently reducing morbidities and mortalities associated with inadequate management of NMFIs.

## Introduction

Malaria is a serious and sometimes life-threatening infectious disease that affects humans and other animals, caused by several species of the Plasmodium parasite and transmitted by the bite of an infected Anopheles mosquito. Malaria typically manifests as a febrile illness that can be fatal if unrecognized, especially in young children. Approximately 3.2 billion people live in malaria endemic areas, and, in 2015, there were an estimated 214 million cases and 438,000 deaths attributable to the disease. Most of these deaths occurred in sub-Saharan African countries in children under five years of age [1]

Febrile illness is fever (defined as temperature >38°C [2]) without localizing features and is among the most common reasons for persons to seek health care in low and middle-income countries [1]. Data from community-based infection prevalence in childhood above 34–37%, show that 50% or more of fevers are likely to be associated with malaria infection [3]. For patients who present with fever without localizing features, clinical diagnosis is difficult and malaria may be the default diagnosis.

In many malaria endemic areas, including Burkina Faso, more than half of these febrile children are considered not to be infected with malaria, as the incidence of this disease is declining due to increased control efforts over the last decade [3–6]. This combined with the guidelines of the World Health Organization (WHO) that recommend confirming a malaria infection in febrile children through a diagnostic test before giving antimalarial treatment [7], prompts the need to search for an alternative cause of fever. This has created a diagnostic dilemma for health workers, by having a large group of patients with so called “unexplained” or “non-malaria” fever and with few or no diagnostic tools available to guide the subsequent management of these febrile cases.

Non-malarial febrile illnesses (NMFI) are infectious diseases affecting patients who show signs of indistinguishable fever thus necessitate use of malaria diagnostic tests, but these tests turn out to be negative for malaria [3]. Laboratory assays for many febrile diseases are often complex, costly, and may have limitations of sensitivity and specificity; and are not widely available in areas where epidemiologic information on the etiology of febrile illness is sparse [4].

NMFI accounts for about half of all fever presenting morbidities among under-five children in sub-Saharan Africa. A study of 70,666 fevers identified across 42 sub-Saharan countries estimated that there were 655.6 million reported fevers in 2007. Malaria did not appear to be the major driver of fever among the children, highlighting the importance of building a clearer picture of causes of NMFI to inform both patient management and prevention efforts. NMFI is known to carry case fatality ratios that equal or exceed malaria in low-resource areas.[4]. Most NMFI among children are caused by bacteria and viruses [5,6]. This implies the need for ‘test before treat’ as opposed to presumptive treatment of fevers as malaria, to avoid unwanted consequences [7]. Many studies have reported cases of prescription of antimalarial medications to febrile under-fives who were negative for malaria [8–10] with some showing that more than 90% of prescriptions for antimalarial drugs in low-moderate transmission settings were for patients for whom a test requested by a clinician was negative for malaria [11]. Despite the presence of quality-assured malarial Rapid Diagnostic Tests (mRDTs) and parasitological tests, children under-five in Africa are often treated with malaria drugs when they do not have malaria, contributing to increased incidences of antimalarial drug resistance, as well as failure to treat the actual underlying causes of fever [8,12]. Antimalarial prescription to patients before carrying out laboratory tests of malarial antigens increases child mortality [9,12,13]. Over-diagnosis and over-treatment of malaria in children under-five poses a threat to the survival of admitted children, especially in the critical 48 hours of admission [5].

In Tanzania, the main clinical diagnoses among febrile under-fives who test negative for malaria include respiratory tract infections, pneumonia and urinary tract infections [9]. In Kenya, symptoms including fever, appetite loss, vomiting, headache and diarrhea were significantly more common among febrile under five children tested negative for malaria than those tested positive. These under-fives were however prescribed antimalarial drugs [14].

Literature supports several factors as predictors for prescription of antimalarial drugs to children tested negative for malaria. Clinical assessment findings, demographic characteristics of the child and in some cases, the healthcare workers characteristics have been associated with treatment type (malarial/non-malarial) of non-malarial febrile illnesses in children aged five years and under [9,14,15]. Odds of prescribing antimalarial medications to inpatient febrile under-fives who were negative for malaria were more than three times as compared to outpatient under-fives [9]. In Kenya, World Health Organization Integrated Management of Childhood Illness (IMCI), danger signs (unable to drink/ breastfeed, vomits everything, convulsions, lethargic/unconscious) and fever lasting ≥7 days and ≥39° C were predictors for prescribing antimalarial drugs [15]. In Nigeria, fever, decreased appetite, and temperatures between 38°C and 41°C were associated with children prescribed with antimalarial drugs [16]. In Tanzania, health care providers working at hospitals significantly increased odds about four-fold to prescribe antimalarial as compared to health care providers working in the dispensaries[9]. Study in Nigeria reported no difference between proportion of male and female febrile children aged less than 60 months who were prescribed antimalarial drugs having tested negative for malaria [17].

### Public health implications

Sustainable Development Goals (SDGs) aim to significantly reduce childhood mortality to less than 25 from 43 per 1,000 live births globally. A major reduction in mortality due to infectious diseases in most low and middle income countries is necessary for the achievement of the SDGs [18]. In Kenya, under-five mortality rate stands at 52 deaths per 1,000 [19]. Childhood malaria has received significant funding and with increased availability of RDTs, it has been shown that confirmed malaria cases are on the decline [20]. However, there is a growing number of NMFI in limited resource settings, especially in Sub-Saharan Africa. Consequently, the limited resource settings do not have the tools to manage the subsequently recognized NMFI which results in incompatibilities between confirmed diagnosis and treatment of the actual diagnoses [21]. Several bacterial, viral and parasitic infections that cause febrile illness have a non-specific clinical presentation, making it difficult for a healthcare worker to distinguish one from another based solely on clinical history taking and performing a physical examination [22]. This study sought to identify the clinical and demographic factors associated with treatment type (malarial/non-malarial) of non-malarial febrile illnesses and treatment outcome, in children aged five years and under, admitted at the Kenyatta National Hospital. This would help inform healthcare providers on the correct management and treatment of non-malarial febrile illnesses in under-fives.

## Materials and methods

### Study site

Kenya sitting astride the equator is located in the eastern region of Sub-Saharan Africa, with a population of approximately 47 million and an under five mortality rate of 52 per 1000 [19]. Four million of these citizens reside in Nairobi, the capital city of Kenya.

Malaria still remains a major public health problem in Kenya and accounts for an estimated 16% of outpatient consultations based on data from the routine health information system [19]. Malaria transmission and infection risk in Kenya is determined largely by altitude, rainfall patterns, and temperature. The two rainy seasons are the long rains occurring from March to May and the short rains from October to December. Temperatures are highest from February to March and lowest from July to August. Therefore, malaria prevalence varies considerably by season and across geographic regions [23].

Prevalence data from a study using cross-sectional data extracted from the Kenya Malaria Indicator Survey (2015) showed that prevalence of malaria in children 10–14 years was 10.22%, while in children under the age of 5 it was 4.83%. Malaria prevalence was also found to be higher among male children (8.23%) than female children (8.04%) [24].

This study was conducted at Kenyatta National Hospital (KNH), the largest public, tertiary referral hospital in Kenya and located in Nairobi, therefore the apex of health system in Kenya. The hospital receives most of the severe referred cases of childhood fevers from across the country. Approximately, 84% of all the children received in Kenyatta Hospital between July and August 2011 presented with fever [25].

### Study design

The study adopted a positivist epistemological approach with a cross-sectional, descriptive study design. The design was appropriate since the study tested the degree of association between and among variables at a specific point in time. This study design determines the status of phenomena without controlling or manipulating variables [26] and is concerned with hypotheses formulation and testing between variables [27].

The primary outcome variable in this study was measured through a proxy variable, which allows for a variable that cannot be confirmed to be included in the model to be estimated [28]. NMFI is loosely defined as all febrile illnesses that do not have malaria as its etiology. With the use of secondary data, these NMFIs cannot be confirmed hence a proxy procedure was conducted in two phases. In the first phase, those who received the antimalarial medication despite whether they had a positive or negative microscopy result were first isolated. The study then zeroed in on those who received the correct antimalarial prescription (antimalarial prescribed for patient with positive microscopy result) in the second phase. This was then used to establish the patients who received antimalarial medication despite having negative microscopy results as incorrectly treated NMFIs.

### Data collection

Medical records of children aged five years and under, admitted with febrile illness from March-August 2017, defined as the long rainy season in Kenya [23] was used. It is estimated that 69% of children that were admitted in county hospitals in western Kenya tested negative for malaria but were treated with malarial drugs [29]. Using Cochran (2007) formula, the number of children to be involved in this study was given by;

n=(Z^2×p×(1−p))/d^2=(〖1.96〗^2×0.69×(1−0.69))/〖0.05〗^2=328.7≈329medicalrecords.

Where Z is the standard normal variate, p is the proportion of children who test negative for malaria and were treated with malaria drugs, n is the desired sample size and d is the margin of error. Nevertheless, all the records during this period were used to eliminate selection bias.

Data was obtained from the health information department at KNH. Data obtained on admission of the febrile patients including; medical history, clinical assessment and treatment was provided by attending medical doctors. Standardized training is provided in Kenyan medical schools on history taking and performance of a physical examination. These are supplemented with management guidelines of various illnesses by the health facilities, Ministry of Health and the WHO. Relevant data to the study including; clinical examinations (recorded as yes or no), diagnostic test results, and demographic data on the child’s sex and age was extracted from patient records and entered electronically into study database using Epi-Data Manager software within the hospital premises. The database was password protected to ensure safety and confidentiality of data. Upon completion of data extraction and entry into Epi-Data Manager, the database file was exported to Statistical Package for Social Sciences (SPSS) version 20 for analysis.

#### Inclusion criteria

Children aged five years and under.

Admission temperature of 38° C and above.

#### Exclusion criteria

Children referred to KNH with a known diagnosis of malaria, for further management.

Children aged five years and under whose medical records had missing information on more than two study variables.

Admission temperature of below 38°C.

### Ethics statement

Ethical approval was obtained from University of Liverpool Research Ethics Committee and Kenyatta National Hospital-University of Nairobi Ethics Research Committee (KNH-UoN, ERC) (P489/08/2017) and authorization provided by the Kenyatta National Hospital Health Information Department before the actual study commenced. The Epi-Data Manager entry template was developed in a way that de-identified the patients during extraction and ensured that none of the information could be linked back to the patients. Data will be kept securely for five years from the time of extraction from hospital.

### Data management and analysis

The data was collected and recorded in two parts, diagnosis and treatment sections. The diagnosis section contained eight, and treatment section had two elements of assessments. The first treatment element of assessment (Was the child correctly prescribed an antimalarial after a positive microscopy result?) was used as proxy to measure the variable of interest which is the children under-five who tested negative for malaria but were treated with antimalarial medication while postulated to be suffering from NMFI.

All the responses to the questions were YES/NO except the age variable which was recorded as numbers. The gender variable was captured as Female/Male.

#### Main variables

The YES/NO responses were coded in SPSS IBM 20 as 1/0 respectively. While gender F/M was coded as 1/2 respectively.

Age variable was recoded into a factor variable because there were cases of < 1 (less than one year) which was not specified in months; hence it was recorded as ordinal data.

#### Other variable

The variable which was indicating whether the patient is deceased or not deceased was created as Deceased/Not deceased and coded as 1/0 respectively.

#### Outcome variables

The primary outcome variable was the children with NMFI treated with antimalarial drugs despite negative microscopy results measured indirectly using correct/incorrect prescription of antimalarial after microscopy results responded to as YES/NO respectively and coded as 1/0 in SPSS.

The secondary outcome Variable was whether the patient was Deceased/Not deceased at the time of discharge and was coded as 0/1 respectively.

To capture the No response and Not Applicable (N/A) the codes assigned to these variables was 99 and 88 respectively.

A total of 341 surveys were numbered from 1 to 341 as per standard rule for data entry. This helped to identify and track the surveys to avoid erroneous entry. The data was then entered to SPSS and screened for errors before being used for analysis.

#### Analysis procedures

The data to assess the primary outcome was rigorously processed using R programming analytic software and SPSS IBM 20. The frequencies of the variables were run using SPSS to check if there were any missing data points as usually reported by the frequency output tables. There were two missing data points on patients’ identification number. This variable would be important in tracing the patients’ records in the database if required but had no effect on the analysis and results.

There were two treatment questions in the data capture sheet; the primary outcome question (Was the child correctly prescribed an antimalarial after a positive microscopy result) serving as the proxy variable and the isolating variable (Is it verified that the child with a negative microscopy result did not receive an antimalarial). Those who answered the first question with a YES/NO the second one was N/A, and vice versa. The data evaluated for the primary outcome was 72 cases (n = 72) and 269 cases (n = 269) for the second outcome variable.

The descriptive statistics was done using SPSS. The descriptive analysis was done in two steps. The frequencies of all the variables were generated and tabulated in the predefined table. The crosstab analysis was generated for specific variables to assess the relationship between the primary outcome and the predictors displayed in the tables.

#### Statistical tests

The data set is categorical data comprising of the YES/NO responses. Male/Female and numerical age variables were transformed to ordinal data type. Non-parametric/categorical chi-square test was used to establish associations between outcome variable and exposure factors. On the other hand, binary logistic regression was used to model the categorical outcome variables against the categorical exposure factors.

Chi square test was done to test the associations between two variables, outcome variable and a predictor (bivariate). The results from the chi square test were recorded in the tables.

Binary logistic regression analysis was conducted using R to assess the association of the predictors and the binary outcome variables. The data was converted to comma separated values (CSV) and exported to R. The results between the primary variable and the predictor’s variables were generated and recorded in the tables. The secondary variable was assessed against all the predictor variables to test if there were any associations. The binary logistic model yielded odd ratios, p-values and confidence intervals (CI) for drawing valid conclusions.

## Results

The study used records from 341 children aged five years and under with fever of 38°C and above. The study findings were categorized into the following themes:

- Demographic characteristics;
- Clinical factors (exposures);
- Treatment outcomes;
- Relationship between outcomes and clinical factors (exposures);
- Relationship between outcomes and demographic characteristics

### Demographic characteristics

**Fig 1:**
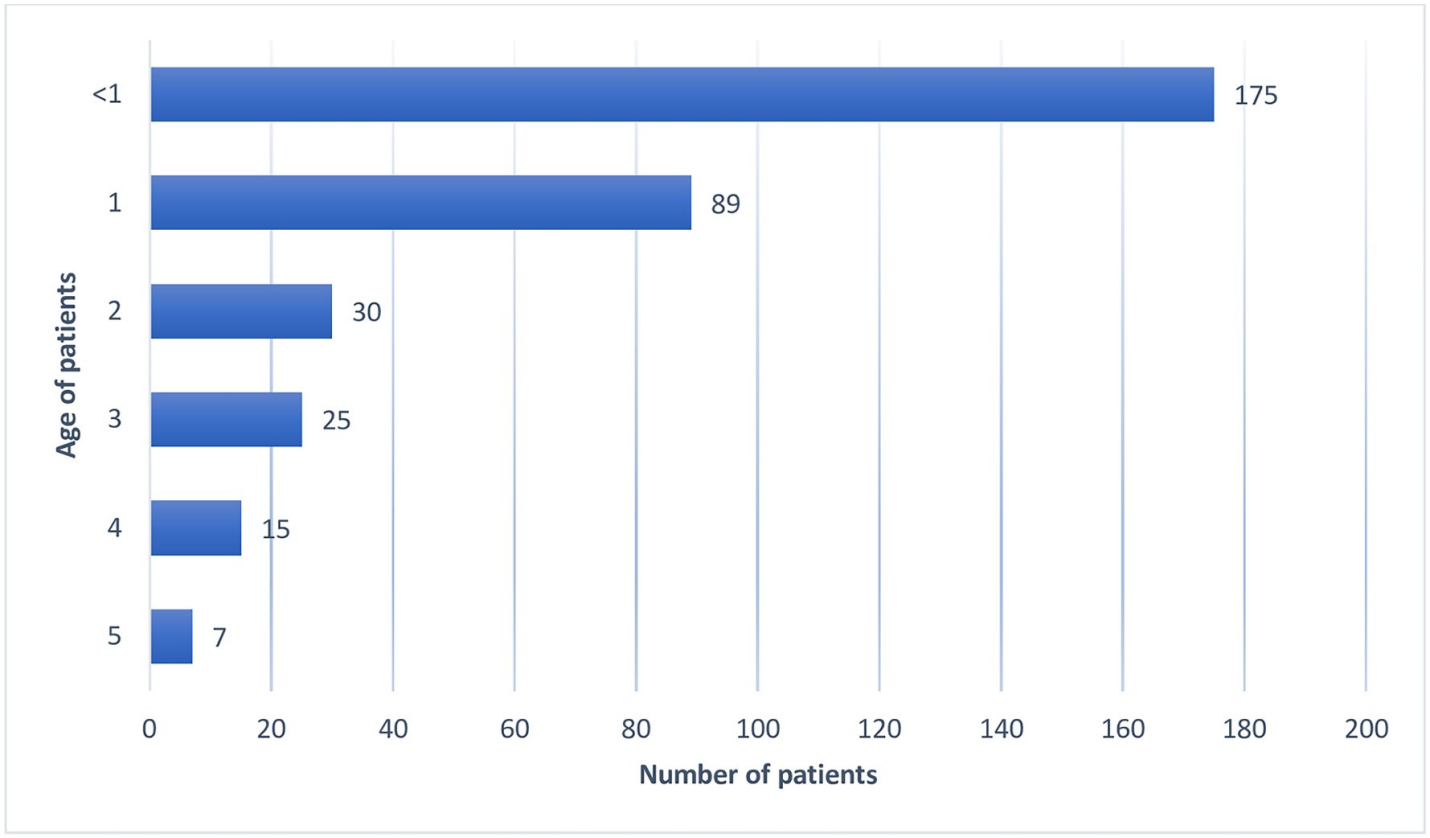
Under-fives admitted at Kenyatta National Hospital with febrile illness from March-August 2017 by age.

Most of the under-five patients (264) in this study who were admitted at the hospital were aged one and below, making 77.48% of the total admission (Fig 1). The study sample comprised of majority males 186 (54.5%) with 155 (45.5%) females.

### Clinical factors (Exposures)

Exposures were reported based on clinical factors that were assessed as indicated in the files by the attending clinician on admission of the patient. Hours since fever onset were noted in 262(76.8%) children. Immunization cards were assessed among 196(57.5%) children. Temperature was taken for 337(98.8%) children. Danger signs which included ‘at least 3 main symptoms (cough, diarrhea, fever ear problem)’ was assessed and noted in 253(74.2%) children. Presence or absence of respiratory distress (stridor, severe chest in-drawing, subcostal retractions or grunting) was noted in 261(76.5%) children. Pallor, jaundice, and sunken eyes were assessed in 320(93.8%) children. Lethargy, prostration, unconsciousness or either irritability and restlessness was assessed among 197(57.8%). (Table 1).

**Table 1:** Clinical factors (Exposures).

Clinical factors (Exposures)	Frequency (n = 341)	Percent (%)
Hours since fever onset noted	262	76.8
Immunization card assessed	196	57.5
Temperature taken	337	98.8
Danger signs indicated	253	74.2
At least 3 main symptoms assessed and noted (cough, diarrhea, fever ear problem)		
Note of presence or absence of respiratory distress (Stridor, severe chest in-drawing, subcostal retractions or grunting)	261	76.5
Child assessed for pallor, jaundice, sunken eyes	320	93.8
Child assessed for lethargy, prostration, unconsciousness or either irritability and restlessness	197	57.8

### Treatment outcome

Treatment outcome was classified into two; primary and secondary outcome. Primary outcome was based on either correct or incorrect treatment used as a proxy to measure NMFI treated with antimalarial drugs. Among the 341 children sampled, a section of 72(21.1%) were prescribed with antimalarial medication. Among the 72 children, 28(8.2%) were correctly prescribed with antimalarial while 44(12.9%) were incorrectly prescribed with an antimalarial despite a negative microscopy result. The group of 44(12.9%) forms the variable of interest measured indirectly by first isolating those who received antimalarial medication (whether or not they had a positive microscopy result), then zeroed in to those who received the correct antimalarial prescription following positive microscopy result. Hence the primary outcome variable of the study was measured through proxy procedure.

The secondary outcome was captured on basis of whether the patient was deceased/Not deceased at the time of discharge. This is a derived variable to describe whether the death of the patient can be linked to the incorrect treatment of the NMFI with antimalarial drugs. A proportion of 14.1% (48/341) of the patients in this study were deceased at the time of discharge (Table 2). Of this proportion deceased, 11.4% (5/44) patients were treated with antimalarial drugs despite negative microscopy results.

**Table 2:** Outcome.

Outcome	Frequency (n = 341)	Percent (%)
***Primary (Treatment)***		
Correct malarial treatment	28	8.2
Incorrect malarial treatment	44	12.9
***Secondary***		
Deceased	48	14.1
Not deceased	293	85.9

### Relationship between outcome and clinical factors (Exposures) (n = 72)

#### Treatment (primary) outcome and clinical factors

Assessment of hours since fever onset; immunization cards; temperature; presence or absence of respiratory distress; pallor, jaundice, and sunken eyes; lethargy, prostration, unconsciousness or either irritability or restlessness were not significantly associated with treatment outcome (p-value >0.05). Assessment of danger signs (cough, diarrhea, fever ear problem) significantly influenced treatment outcome (p-value = 0.004), see Table 3.

**Table 3:** Treatment outcome and clinical factors.

Clinical Variables	Treatment outcome (n)		P-value	Bivariate OR (95% CI)	P-value	Multivariate OR (95% CI)
	C	I				
Hours since fever onset noted.	(28)	(44)	0.323	0.51 (0.12,2.12)	0.312	0.37(0.05,2.64)
Immunization card assessed.			0.340	0.71(0.27,1.88)	0.334	0.52(0.13,2)
Temperature taken.			0.996	NA	0.061	NA
Danger signs indicated*.			0.103	2.11(0.76,5.84)	0.089	3.42(0.78,14.97)
Note of presence or absence of respiratory distress**			0.008	0.49(0.17,1.41)	0.004	0.13(0.03,0.58)
General physical exam done***.			0.995	NA	0.261	NA
Neurological exam done ****			0.419	0.63(0.23,1.72)	0.415	1.79(0.43,7.41)

C- Correct; I- Incorrect; OR- Odds ratio; CI- Confidence interval

* At least 3 symptoms associated with febrile illness indicated in notes- Cough, diarrhea, fever, ear problem

** Signs of respiratory distress- Stridor, severe chest in-drawing, subcostal retractions or grunting

***Child assessed for pallor, jaundice, sunken eyes

****Child assessed for lethargy, prostration, unconsciousness or either irritability and restlessness.

#### Respiratory distress and antimalarial prescription

The prescription of antimalarial drugs despite negative microscopy results was significantly associated with whether the patient was checked on presence or absence of respiratory distress (X-square = 7.1598, P value = 0.0278). This implied that assessment of presence or absence of respiratory distress, influenced if the NMFI patient received antimalarial prescription even after negative microscopy results, see Table 4. The patient whose respiratory status was checked based on (stridor, severe chest in-drawing, subcostal retractions or grunting) was significantly associated with antimalarial treatment after negative microscopy results (P value = 0.008, 95%CI (1.71, 37.07)). The patient whose respiratory status was checked was 0.13 CI (0.03,0.58) times less likely to receive antimalarial drugs after a negative microscopy result than one whose respiratory status was not checked.

**Table 4:** Respiratory distress and antimalarial prescription.

		Was the child correctly prescribed an antimalarial after a positive microscopy result?			Total
		NO	YES	Not Applicable	
Are there notes to indicate presence or absence of respiratory distress (Stridor, severe chest in-drawing, subcostal retractions or grunting)? If less than 3 indicate No.	NO	18	7	55	80
	YES	26	21	214	261
Total		44	28	269	341

#### Mortality (secondary outcome) and clinical factors

There was potentially good survival chance for the patients who were checked for presence or absence of respiratory distress (proportion of respiratory distress checked and survived (223/341) = 0.654 Vis a Vis proportion of those whose respiratory distress not checked and survived (70/341) = 0.205). This meant that 1 in every 5 patients checked for respiratory distress survived. Patients who were assessed for danger signs with main symptoms of (cough, diarrhea, fever, ear problem) were 3.15 times more likely to survive than those who were not assessed (OR = 3.15, P value = 0.007, 95%CI (1.38, 7.18).

### Relationship between treatment outcome and demographic factors (n = 72)

#### Primary treatment outcome and demographic factors

Gender and age significantly influenced the primary NMFI treatment outcome (p-values <0.05), see Table 5.

**Table 5:** Treatment outcome and demographic variables.

Demographic Variables	Treatment outcome		P-value	Bivariate OR (95% CI)	P-value	Multivariate OR (95% CI)
	C	I				
Gender						
Male	16	19		0.51(0.19,1.34)	0.024	0.21(0.05, 0.89)
Female	12	25				
Age groups						
<1 Reference age group (p-value = 0.002)						
1	9	11		0.07(0.01,0.68)	0.015	0.05 (0,0.55)
2	8	3		0.03(0,0.28)	0.001	0.01 (0,0.17)
3	6	8		0.09(0.01,0.87)	0.039	0.07(0.01,0.88)
4	4	6		0.1 (0.01,1.09)	0.071	0.09(0.01,1.22)
5	N/A					

C- Correct; I- Incorrect; OR- Odds ratio; CI- Confidence interval **(n = 72)**

#### Gender and prescription of antimalarial drugs after negative microscopy result

The gender of the patients was significantly associated with prescription of antimalarial treatment after negative microscopy results (P value = 0.024, OR = 0.21, CI (0.05, 0.89)). Data revealed that a male patient was 0.21 times less likely to receive an antimalarial after negative microscopy results compared to female patients, see Table 5. The manner in which male and female patients received antimalarial treatment after negative microscopy was not significantly different (P value = 0.267), see Table 6.

**Table 6:** Gender and correct prescription of the antimalarial drugs.

		Was the child correctly prescribed an antimalarial after a positive microscopy result?			Total
		NO	YES	Not Applicable	
Patient’s Gender	Female	25	12	118	155
	Male	19	16	151	186
Total		44	28	269	341

#### Age and prescription of antimalarial drugs after positive microscopy result

The age of the patients was significantly associated with prescription of antimalarial treatment after negative microscopy results (X-squared = 10.221, df = 4, P value = 0.03686), see Table 7.

**Table 7:** Age, demographics and prescription of the antimalarial drugs.

		Was the child correctly prescribed an antimalarial after a positive microscopy result?			P-Values (from multi variate)
		NO	YES	Total	
What is the age of the patient at assessment?	**1 year**	11	9	89	**0.015**
	**2 years**	3	8	30	**0.001**
	**3 years**	8	6	25	**0.039**
	**4 years**	6	4	15	**(0.071)**
	**5 years**	0	0	7	**-**
	**<1 year**	16	1	175	**Reference age**
Total		44	28	341	

With reference to patients aged under 1 year (< 1 year), a patient aged 1 year was 0.05 times less likely to receive antimalarial treatment after negative microscopy results, those aged 2 years were 0.01 times less likely to receive antimalarial treatment after positive microscopy results, aged 3 years were 0.07 times less likely to receive antimalarial treatment after negative microscopy results while those aged 4 years were 0.09 times less likely to receive correct anti-malarial treatment after negative microscopy results.

#### Secondary outcome and demographic factors (n = 72)

**Gender and mortality**. More females (29) than males (19) succumbed to death in under-five patients at KNH during this period of study. The gender of the patients was significantly associated with the mortality (P value = 0.025).

### Summary

The study sample comprised majority males and children aged one year and under.

Majority of the clinical factors (exposures) were assessed at admission, including; hours since fever onset, immunization cards, temperature, danger signs, respiratory distress, pallor, jaundice and sunken eyes, and lethargy, prostration, unconsciousness or either irritability and restlessness.

Outcomes included; 28(8.2%) of the children were correctly prescribed an antimalarial after positive microscopy results, while 44(12.9%) were prescribed an antimalarial despite a negative microscopy result. Mortality was the secondary outcome and was reported among 14.1% of the children.

Among the clinical factors; assessment of presence or absence of respiratory distress significantly affected treatment of patients with a negative microscopy with an antimalarial, as it decreased the prescription of an antimalarial to those with a negative microscopy by 0.13 times. Assessment of danger signs was also noted to increase the chances of survival by 3.15 times compared to those who were not assessed.

Demographic factors, gender and age significantly influenced the primary treatment outcome. A male patient was 0.21 times less likely to receive antimalarial treatment after negative microscopy result compared to female patients. Patients aged <1 year were more likely to receive antimalarial treatment after negative microscopy results compared to those aged ≥1 year. Children aged 2 years were less likely to receive antimalarial treatment after negative microscopy results compared to ages 1,3 and 4.

It was also noted that a significant number of females succumbed to death than males.

## Discussion

### Implications of the findings

#### Incorrect prescription of antimalarial drugs for treatment of NMFIs

Of the 72 children in this study who were prescribed an antimalarial drug, 12.9% (44) had laboratory and treatment reports in their medical records that indicated that they had been prescribed with an antimalarial medication despite testing negative for malaria according to the microscopic examination.

The prescription of antimalarial medication despite negative microscopy results is similar to other studies that reported cases of prescription of antimalarial medications to febrile under-fives who were negative for malaria. A study in Lagos, Nigeria [8] reported over diagnosis of malaria and subsequent presumptive treatment in the under-five group. Out of the 1028 0-≤5 years old children tested microscopically for malaria only 174 (16.9%) tested positive, though all the children received antimalarial as per IMCI guidelines. This gave an overtreatment of 83.1% which is quite high compared to this study’s overtreatment of 12.9%, though a smaller sample size was used in this study and the areas have different malaria endemicity. Almost similar rates were found in a study on prescription practices for NMFI among under-fives in the Lake Zone, Tanzania. Over prescription of antimalarial drugs was at 12% similar to this study’s 12.9%, while 14% of under-fives had a correct diagnosis of malaria and prescribed an antimalarial which was slightly higher than this study’s 8.2% [9]. In south eastern Nigeria a study also demonstrated an overtreatment of 72.1% with antimalarial medication in a study population of 560 children. These children were also prescribed an antimalarial drug despite testing negative for malaria [30]. Incorrect treatment of NMFIs with antimalarial drugs is likely to contribute to increased incidences of antimalarial drug resistance as well as failure to treat the actual underlying causes of fever resulting in increasing morbidities and even mortalities contributing to increased patient costs [8,12]. Current WHO guidelines recommend parasitological diagnosis before prescription of antimalarial, but from this study and others, there seems to be a lack of confidence by the prescribing health care workers on the microscopy results received, together with non-adherence to the guidelines. This could be brought about by their previous adherence to the IMCI guidelines on treating all febrile patients with antimalarial medication and reliance on clinical rather than laboratory diagnosis.

#### Incorrect NMFI treatment and mortality

Early detection of children at the highest risk of dying and providing the correct diagnosis and early treatment, aids in the prevention of further morbidities and even mortalities. The clinical signs and symptoms of malaria can be confused with numerous other febrile causing etiologies such as pneumonia, typhoid, dengue, chikungunya and other bacterial and viral infections. Over-diagnosis and overtreatment of malaria leads to under diagnosis of NMFI. Lack of proper NMFI treatment may lead to increased morbidities and could have contributed to the 11.4% mortalities in the group that were prescribed an antimalarial despite negative microscopy in this study. A study in a tertiary referral center in Ghana did prospective data collection followed by retrospective analysis of blood culture. The findings concluded that patients who presented with WHO signs and symptoms of severe malaria but had a negative microscopy results had high mortality due to a higher risk of bacterial sepsis [13].

#### Clinical and demographic factors associated with treatment with antimalarial drugs

Several factors have been reported to predict the prescription of antimalarial drugs to children despite testing negative for malaria. Children who have been established to show signs and symptoms of severe disease are more likely to be prescribed an antimalarial despite negative malaria test results as the clinicians try to avoid an opportunity to prevent further morbidity or mortality due to unavailability of diagnostics that could correctly identify the NMFI [15]. A demographic survey and malaria surveillance study in the Kenyan highlands [14], demonstrated that fever and headache were common clinical symptoms for presumptive treatment with an antimalarial in under-five children. In a cross-sectional hospital based study in Sudan, apart from the clinicians’ request for a malaria test, there were no clinical or demographic factors associated with the clinicians’ antimalarial prescription [31]. In a study conducted in Western Kenya, assessment of ≥1 IMCI danger signs increased the chances of antimalarial treatment [15]. A cross-sectional observational study of under-five children conducted in Borno state Nigeria also provided contrasting results, reporting that the probability of incorrectly prescribing an antimalarial to NMFI patients based on empirical clinical diagnosis was at 71%, with no specific factors significantly associated. Demographic factors also did not show any difference between proportion of male and female febrile children aged less than 60 months who were prescribed antimalarial drugs having tested negative for malaria [17]. The differences in the conclusions observed could be due to the different populations assessed and malaria endemicity of the regions studied.

### Strengths of the study

Use of a cross-sectional study enabled the study of multiple outcomes and exposures and adequately answered the generated hypotheses.

The study assessed the patients’ outcome data which was included in the secondary outcome variable as deceased or not deceased. It revealed a mortality rate of 14.1%, with 11.4% of these total mortalities being from the group that was incorrectly prescribed an antimalarial despite negative microscopy. This provides a better understanding on the need to provide correct diagnosis and treatment of NMFIs, to achieve the SDGs on reducing infant mortality rate.

### Limitations of the study

The study is not able to look at the reasons behind the health practitioners prescribing antimalarial medication to patients with negative microscopy tests. This would have been better achieved with a qualitative study.

Factors such as the level of training of the clinicians, supervision, the laboratory capacity, expectations of the patients were not taken in account which could all possibly affect the prescription practices.

The study was done at Kenyatta National Hospital, one of the tertiary referral hospitals in Kenya hence it receives many sick children, limiting the generalizability of the findings to the rest of the Kenya.

The study did not collect any data on previous use of antimalarial or antibiotic medications before referral to KNH, and this could be a major influence on the clinicians’ prescription practice.

## Conclusion

This study supports that clinical and demographic factors such as age, gender and assessment of presence or absence of respiratory distress are predictors of prescription of antimalarial drugs in under-fives. Tackling the NMFI challenge requires a concerted effort, to eliminate incorrect treatment with antimalarial medication, while ensuring correct diagnosis and treatment of the specific illness occurs.

It has been repeatedly showcased that inadequate management of NMFIs, is a major cause of morbidities and mortalities among under-fives, especially in Sub-Saharan Africa. This study has shown that clinical and demographic factors play a role in treatment type of NMFI and survival outcomes; with a proportion of the 14.1% of the mortalities in this study postulated to be associated with delayed diagnosis and incorrect treatment of NMFI. There is a need for further exploration to ascertain factors that affect treatment type, together with factors that influence the prescribing physicians, and their knowledge and attitudes on NMFI treatment.

Adherence to diagnostic and treatment guidelines in management of febrile children, will assist in the delineation of non-malaria febrile illnesses. This brings about a subsequent need to create and implement guidelines on the correct diagnosis and management of these NMFIs.

## Supporting information

S1 Appendix Non-malarial febrile illness clinical assessment questionnaire. (PDF)

## References

[1] World Health Organization. World Malaria Report 2015 [Internet]. 2015. https://apps.who.int/iris/bitstream/handle/10665/200018/9789241565158_eng.pdf;jsessionid=989E947C0C02AA8D81D43E5E83B0D934?sequence=1

[2] Elshout G, Monteny M, van der Wouden JC, Koes BW, Berger MY. Duration of fever and serious bacterial infections in children: a systematic review. BMC Fam Pract. 2011;12 10.1186/1471-2296-12-33 21575193 PMC3111584

[3] Okiro EA, Snow RW. The relationship between reported fever and Plasmodium falciparum infection in African children. Malar J. 2010;9 10.1186/1475-2875-9-99 20398428 PMC2867992

[4] Gething PW, Kirui VC, Alegana VA, Okiro EA, Noor AM, Snow RW. Estimating the Number of Paediatric Fevers Associated with Malaria Infection Presenting to Africa’s Public Health Sector in 2007. Whitty CJM, editor. PLoS Med. 2010;7: e1000301 10.1371/journal.pmed.1000301 20625548 PMC2897768

[5] Reyburn H. Overdiagnosis of malaria in patients with severe febrile illness in Tanzania: a prospective study. BMJ. 2004;329: 1212–0. 10.1136/bmj.38251.658229.55 15542534 PMC529364

[6] Pondei K, Kunle-Olowu OE, Peterside O. The aetiology of non-malarial febrile illness in children in the malaria-endemic Niger Delta Region of Nigeria. Asian Pac J Trop Dis. 2013;3: 56–60. 10.1016/S2222-1808(13)60012-2

[7] D’Acremont V, Bosman A. WHO Informal Consultation on Fever Management in Peripheral Health Care Settings: A Global Review of Evidence and Practice [Internet]. World Health Organization; 2013. http://www.who.int/malaria/mpac/who_consultation_fever_management_briefing.pdf

[8] Oladosu OO, Oyibo WA. Overdiagnosis and Overtreatment of Malaria in Children That Presented with Fever in Lagos, Nigeria. ISRN Infect Dis. 2013;2013: 1–6. 10.5402/2013/914675

[9] Kazaura M, Lugangira K, Kalokola F. Prescription practices for non-malaria febrile illnesses among under-fives in the Lake Zone, Tanzania. Asian Pac J Trop Dis. 2016;6: 759–764. 10.1016/S2222-1808(16)61125-8

[10] Okoro CI, Chukwuocha UM, Nwakwuo GC, Ukaga CN. Presumptive Diagnosis and Treatment of Malaria in Febrile Children in Parts of South Eastern Nigeria. J Infect Dis Ther. 2015;03 10.4172/2332-0877.1000240

[11] Reyburn H, Mbakilwa H, Mwangi R, Mwerinde O, Olomi R, Drakeley C, et al Rapid diagnostic tests compared with malaria microscopy for guiding outpatient treatment of febrile illness in Tanzania: randomised trial. BMJ. 2007;334: 403–403. 10.1136/bmj.39073.496829.AE 17259188 PMC1804187

[12] Onchiri FM, Pavlinac PB, Singa BO, Naulikha JM, Odundo EA, Farquhar C, et al Frequency and correlates of malaria over-treatment in areas of differing malaria transmission: a cross-sectional study in rural Western Kenya. Malar J. 2015;14: 97 10.1186/s12936-015-0613-7 25890202 PMC4349314

[13] Evans JA, Adusei A, Timmann C, May J, Mack D, Agbenyega T, et al High mortality of infant bacteraemia clinically indistinguishable from severe malaria. QJM. 2004;97: 591–597. 10.1093/qjmed/hch093 15317928

[14] Mutanda AL, Cheruiyot P, Hodges JS, Ayodo G, Odero W, John CC. Sensitivity of fever for diagnosis of clinical malaria in a Kenyan area of unstable, low malaria transmission. Malar J. 2014;13: 163 10.1186/1475-2875-13-163 24885660 PMC4021053

[15] Onchiri FM. ‘Changing Etiologies of Febrile Illness in Areas of Differing Malaria Transmission in Rural Kenya. University of Washington 2014.

[16] Abdulkadir I, Rufai H, Ochapa S, Malam M, Garba B, Oloko AG, et al Malaria rapid diagnostic test in children: The Zamfara, Nigeria experience. Niger Med J. 2015;56: 278 10.4103/0300-1652.169744 26759514 PMC4697217

[17] Elechi H, Rabasa A, Alhaji M, Bashir M, Bukar L, Askira U. Predictive indices of empirical clinical diagnosis of malaria among under-five febrile children attending paediatric outpatient clinic. Ann Trop Med Public Health. 2015;8: 28 10.4103/1755-6783.157275

[18] United Nations. The Sustainable Development Goals Report 2016. United Nations, Geneva; 2016.

[19] Kenya National Bureau of Statistics [KNBS]. Kenya Demographic and Health Survey 2014. KNBS, MoH, NACC, KEMRI & NCPD, Nairobi; 2015.

[20] Acestor N, Cooksey R, Newton PN, Ménard D, Guerin PJ, Nakagawa J, et al Mapping the Aetiology of Non-Malarial Febrile Illness in Southeast Asia through a Systematic Review—Terra Incognita Impairing Treatment Policies. Bassat Q, editor. PLoS ONE. 2012;7: e44269 10.1371/journal.pone.0044269 22970193 PMC3435412

[21] Crump JA, Morrissey AB, Nicholson WL, Massung RF, Stoddard RA, Galloway RL, et al Etiology of Severe Non-malaria Febrile Illness in Northern Tanzania: A Prospective Cohort Study. Picardeau M, editor. PLoS Negl Trop Dis. 2013;7: e2324 10.1371/journal.pntd.0002324 23875053 PMC3715424

[22] Crump JA, Kirk MD. Estimating the Burden of Febrile Illnesses. Torgerson PR, editor. PLoS Negl Trop Dis. 2015;9: e0004040 10.1371/journal.pntd.0004040 26633014 PMC4668833

[23] Kenya Meteorological Department. The Outlook for the October-November-December (OND) 2017 Season and Review of Rainfall During the “Long Rains” (March to May) 2017 & June-July-August (JJA) 2017 Seasons [Internet]. Ministry of Environment and Natural Resources State Department of Environment; http://www.meteo.go.ke/pdf/seasonal.pdf

[24] Sultana M, Sheikh N, Mahumud RA, Jahir T, Islam Z, Sarker AR. Prevalence and associated determinants of malaria parasites among Kenyan children. Trop Med Health. 2017;45: 25 10.1186/s41182-017-0066-5 29085254 PMC5651573

[25] Wasilwa IM. Profiles of Children Referred To Kenyatta National Hospital with Acute IMCI Listed Illnesses. University of Nairobi 2012.

[26] Clive H. Private Participation in Infrastructure in Developing Countries: Trends, Impacts, and Policy Lessons. World Bank; 2006.

[27] Best J, Khan J. Research in Education. Prentice Hall of India, New Delhi; 2007.

[28] Diez Roux AV. The Study of Group-Level Factors in Epidemiology: Rethinking Variables, Study Designs, and Analytical Approaches. Epidemiol Rev. 2004;26: 104–111. 10.1093/epirev/mxh006 15234951

[29] Amboko BI, Ayieko P, Ogero M, Julius T, Irimu G, English M, et al Malaria investigation and treatment of children admitted to county hospitals in western Kenya. Malar J. 2016;15: 506 10.1186/s12936-016-1553-6 27756388 PMC5069818

[30] Chukwuocha UM O C, Ukaga CN N G. Presumptive Diagnosis and Treatment of Malaria in Febrile Children in Parts of South Eastern Nigeria. J Infect Dis Ther. 2015;03 10.4172/2332-0877.1000240

[31] Bilal JA, Gasim GI, Abdien MT, Elmardi KA, Malik EM, Adam I. Poor adherence to the malaria management protocol among health workers attending under-five year old febrile children at Omdurman Hospital, Sudan. Malar J. 2015;14: 34 10.1186/s12936-015-0575-9 25627166 PMC4318364

