# Supplementary material for: Factors associated with treatment type of non-malarial febrile illnesses in under-fives at Kenyatta National Hospital in Nairobi, Kenya
Source: PLoS One. 2019 Jun 13;14(6):e0217980. doi: 10.1371/journal.pone.0217980 (PMC6563973; doi:10.1371/journal.pone.0217980)
Supplement: S1 Appendix — (PDF) [file pone.0217980.s001.pdf]

Non-Malarial Febrile Illness Clinical Assessment Questionnaire

A. DIAGNOSIS

(Indicate Yes/No in box if Assessment element is noted in the medical records. Circle whether patient is M/F)

**Assessment Element**	**Patient M/F**
1. What is the age of the patient at assessment? (<1, 1, 2, 3, 4, 5)	
2. Are there notes to indicate the time since onset of fever was noted?	
3. Are there notes to indicate if childhood immunization card was assessed?	
4. Are there notes to indicate if temperature was taken?<br>(If no, or <38°C then exclude)	
5. Are there notes to indicate if danger signs were assessed?<br>At least 3 main symptoms assessed and noted (cough, diarrhoea, fever, ear problem). If less than 3 indicate No.	
6. Are there notes to indicate presence or absence of respiratory distress (Stridor, severe chest in-drawing, subcostal retractions or grunting)?<br>If less than 3 indicate No.	
7. Are there notes to indicate child was assessed for pallor, jaundice, oedema, sunken eyes or dehydration? If less than 3 indicate No.	
8. Are there notes to indicate child was assessed for lethargy, prostration, unconsciousness or either irritability and restlessness?	

B. TREATMENT

**Assessment Element**	
1. Was the child correctly prescribed an antimalarial after a positive microscopy result?	
2. Is it verified that the child with a negative microscopy result did not receive an antimalarial?	

C. OUTCOME

1. Is the patient deceased at the time of discharge?	
